# Assessment of Dietary Supplementation of *Lactobacillus rhamnosus* Probiotic on Growth Performance and Disease Resistance in *Oreochromis niloticus*

**DOI:** 10.3390/microorganisms11061423

**Published:** 2023-05-27

**Authors:** Iqra Noshair, Zakia Kanwal, Ghazala Jabeen, Mateen Arshad, Fakhar-Un-Nisa Yunus, Ramsha Hafeez, Rida Mairaj, Imran Haider, Naushad Ahmad, Suliman Yousef Alomar

**Affiliations:** 1 Department of Zoology, Faculty of Natural Sciences, Lahore College for Women University, Lahore 54000, Pakistan; iqra.noshair@lcwu.edu.pk (I.N.);; 2 Swammerdam Institute for Life Sciences, University of Amsterdam, 1012 Amsterdam, The Netherlands; i.haider@uva.nl; 3 Department of Chemistry, College of Science, King Saud University, Riyadh 11451, Saudi Arabia; anaushad@ksu.edu.sa; 4 Zoology Department, College of Science, King Saud University, Riyadh 11451, Saudi Arabia; syalomar@ksu.edu.sa

**Keywords:** probiotics, *Lactobacillus rhamnosus*, *Aeromonas hydrophila*, *Oreochromis niloticus*, growth performance, disease resistance

## Abstract

Probiotics play a significant role in aquaculture by improving the growth, health, and survival rate of fish against pathogenic organisms. In the present study, we have evaluated the effects of a *Lactobacillus rhamnosus* (*L. rhamnosus*) probiotic on growth performance and disease resistance in *Oreochromis niloticus* (*O. niloticus*) fingerlings. Four different concentrations of *L. rhamnosus* (T1: 0.5 × 10^10^, T2: 1 × 10^10^, T3: 1.5 × 10^10^, and T4: 2 × 10^10^ CFU/kg feed) were administered to fish over a period of three months. *L. rhamnosus* treated fish revealed a high growth increment as compared to the control, and the values of macromolecules (amino acids, fatty acids, and carbohydrates) varied significantly among the treated and control groups. Levels of thyroid hormones were noted to be high in the probiotic-treated groups. A challenge assay was performed with *Aeromonas hydrophila* (*A. hydrophila*). The optimum calculated concentration of probiotics from the growth assay (1.5 × 10^10^ CFU/kg feed) was used for the challenge assay. Fish were divided into four groups as follows: control (Con), probiotic-treated (PL), infected (I), and infected + probiotic-treated (I + PL) groups. Significant variations in hematological parameters were observed among control and treated groups. Histopathological changes were recorded in infected fish, while the infected + probiotic-treated group showed less deformations indicating the positive effect of the probiotic supplementation. The survival rate of fish was also better in the probiotic-treated group. Based on these findings, we conclude that probiotic supplementation enhances the growth and improves immunity of *O. niloticus*. Therefore, we propose that probiotics can be used as promising feed supplements for promoting fish production and disease resistance in aquaculture.

## 1. Introduction

Diseases caused by various fish pathogens have hampered aquaculture productivity and have also affected the growth of aquatic species [1]. Bacterial infections are well-known to cause massive fish mortalities [2]. Aquatic species are frequently targeted by the bacterial pathogen *Aeromonas hydrophila* (*A. hydrophila)*, especially when they are exposed to stressful factors such as water pollution [3]. *A. hydrophila* infection is becoming more common in cultured organisms as a result of increasing pathogen resistance caused by the overuse of antibiotics, which pose serious risks to fish farming [4]. Adding to the further risk posed by *A. hydrophila* is the fact that this bacterium can live in both aerobic and anaerobic conditions as well as in fresh or brackish waters [5]. In fish, *A. hydrophila* causes significant mortalities that result in hemorrhagic illness, ulcerative syndrome, and motile *Aeromonas* septicemia [6].

Probiotics are referred to as live microbial feed additives that penetrate into the host gastrointestinal system and enhance the stability of the gut microbiome, which improves the host’s capacity for growth and disease tolerance [2,7]. Additionally, in comparison to the use of chemical and antibacterial treatments, its benefits stimulate production in a safe and long-term manner [8]. Probiotics are successfully utilized in aquaculture, and they have been reported to enhance survival, immunity, and weight gain [9,10]. Probiotics such as *Lactobacillus* sp., *Saccharomyces* sp., and *Bacillus* sp. are most frequently used in aquaculture, owing to their beneficial effects on fish health through their actions on the intestinal bacteria [11,12].

Fish growth and development appear to be significantly regulated by growth hormone (GH) [13]. Growth hormone acts as a mediator between extrinsic factors that influence development and growth, such as nutrition [14]. Few studies are available about the relationship between probiotics and the production of fish hormones, e.g., growth and thyroid. Thyroid hormones are crucial for a number of physiological processes in the body. The effective functioning of immunological responses under stress depends profoundly upon thyroid hormones [15,16].

Hematological evaluation is an important aspect of fish culture because it allows effective monitoring of fish health [17]. The blood parameters of fish, like those of warm-blooded animals, give specific indications of any changes occurring in the body as a result of injury to organs or tissues associated with infectious diseases [18]. Researchers have focused their attention on the hematological properties of fish in an effort to develop a standard value range because any deviation from it would indicate a disturbance in the physiological process [19]. Fish hematology may be affected by a number of variables, including species, sex, stress, and environment, and by physiological and nutritional status [20].

Histopathological studies have been employed as biomarkers in the evaluation of internal (nutrition) and external (aqueous environment) conditions [21]. Fish undergo histopathological alterations, after being exposed to various toxins and infections, which are frequently employed as indicators to determine the health of the fish [22]. The ability to analyze different organs, such as the kidney, gill, and liver, which carry out crucial physiological processes such as the deposition and bio-magnification of pollutants as well as excretion, is one of the key advantages of employing histological assessment [23]. Moreover, histological studies offer details on diet quality and metabolism in addition to revealing a physiological condition [24].

The aim of the present study was to investigate the effect of dietary supplementation of *Lactobacillus rhamnosus* (*L. rhamnosus*) probiotic on growth performance and disease resistance in *Oreochromis niloticus* (*O. niloticus*). Our hypothesis was that *L. rhamnosus* can enhance the growth of *O. niloticus* and effectively helps in fighting against the *A. hydrophila* challenge. *O. niloticus* is a highly nutritious and affordable source of protein for millions of people, particularly in developing countries. To the best of our knowledge, no investigation has been made to find the effect of *L. rhamnosus* on *O. niloticus*. The findings of this study will be useful for establishing an informative niche about the positive role of *L. rhamnosus* on *O. niloticus* and other related species, which will further help in flourishing the aquaculture sector.

## 2. Materials and Methods

### 2.1. Experimental Fish

Healthy fingerlings of *O. niloticus*, with an approximate weight of 20 ± 2.0 g and length of 10 ± 0.3 cm, were obtained from the Fisheries Training and Research Institute Manawa, Lahore, and transported to the lab in well-aerated polyethylene bags under optimum pH (6.9 to 7.4), temperature (26 to 28 °C), and dissolved oxygen (5.7 to 7.5 mg/L). The length and weight of individual fingerlings were measured, and they were immediately transferred to fiber tanks containing well-aerated tap water. Physicochemical parameters of water were measured (ProQuatro, XA00088-02 YSI, OH, USA) during the treatment period as follows: pH 6.9 to 7.4, temperature range 26 to 28 °C, ammonia 0.1–0.27 mg/L, and dissolved oxygen 5.7 to 7.5 mg/L. Fish were acclimatized for two weeks under laboratory conditions. Fish feed (Hi-Tech Aqua Feeds, Pvt. Ltd., Gujranwala, Pakistan) was given to fish twice a day, in the morning and afternoon, at 2% of the average fish weight. The feed composition was as follows: crude protein (30%), fat (3.5%), moisture (10%), ash (20.8%), and crude fiber (7.5%). The experiment was executed in accordance with the approval obtained from the ethics committee of the Department of Zoology, LCWU, Lahore, via RERC No. LCWU/Zoo/577(d).

### 2.2. Probiotic Treatment

Commercial probiotic ‘Prepro’ (Matrix, Lahore, Pakistan) containing 0.5 × 10^10^ billion CFU of *L. rhamnosus* was used in this study. The probiotic was in dry form and administered in pelleted feed mixed through a mixer. The probiotic was supplemented with the basal diet (above mentioned) in four different proportions: 0.5 × 10^10^, 1 × 10^10^, 1.5 × 10^10^, and 2 × 10^10^ CFU/kg feed. Fish were randomly divided into five groups (*n* = 10 per group) in triplicate experiments (control group (T0): fish given only basal diet and treatment (T1: 0.5 × 10^10^; T2: 1 × 10^10^, T3: 1.5 × 10^10^, and T4: 2 × 10^10^ CFU/kg feed). The total duration of the experimental study was three months.

### 2.3. Growth Performance and Feed Efficiency

Growth was monitored with respect to body mass and body length. The weight and length of each fish in all five groups were recorded at the start and fortnightly during the experimental period. Growth parameters were calculated using the following formulas [25]:

(1) $$\mathrm{Weight}\;\mathrm{gain}=\mathrm{Final}\;\mathrm{weight}\;\left(g\right)-\;\mathrm{initial}\;\mathrm{weight}\;\left(g\right)$$

(2) $$\mathrm{Length}\;\mathrm{gain}=\mathrm{Final}\;\mathrm{length}\;\left(\mathrm{cm}\right)-\;\mathrm{initial}\;\mathrm{length}\;\left(\mathrm{cm}\right)\;$$

(3) $$\mathrm{Specific}\;\mathrm{growth}\;\mathrm{rate}=\frac{\left(\mathrm{Log}\;\mathrm{final}\;\mathrm{weight}\;-\;\mathrm{Log}\;\mathrm{initial}\;\mathrm{weight}\right)\;}{\mathrm{Days}\;}\times 100\;$$

(4) $$\mathrm{Feed}\;\mathrm{conversion}\;\mathrm{ratio}=\frac{\;\mathrm{Feed}\;\mathrm{consumed}\;\left(g\right)\;}{\mathrm{Weight}\;\mathrm{gain}\;\left(g\right)\;}\;$$

(5) $$\mathrm{Protein}\;\mathrm{efficiency}\;\mathrm{ratio}=\frac{\mathrm{Weight}\;\mathrm{gain}\;\left(g\right)\;}{\mathrm{Protein}\;\mathrm{intake}\;\left(g\right)\;}\times 100\;$$

(6) $$\mathrm{Condition}\;\mathrm{factor}=\frac{\mathrm{Body}\;\mathrm{Weight}\;\left(W\right)\;}{\mathrm{Standard}\;\mathrm{length}\;\left(L3\right)\;}\times 100\;$$

### 2.4. Proximate Analysis

At the end of the trial, 5 fish from each group were randomly selected, transferred to a separate tank, and anesthetized by using clove oil (100 μg/L for 40 to 60 s). After the anesthesia, the fish were sacrificed for blood and tissue samples’ collection. Crude protein, moisture, ash, and crude lipid were determined in the treated fish and compared with the body composition of the control group. Samples were oven-dried at 105 °C overnight until the weight was constant for the determination of moisture. They were burned in a muffle furnace at 550 °C for five hours for the determination of ash content. A Kjeldahl protein analyzer (UDK 159, Usmate Velate, Italy) was used to measure the crude protein (N × 6.25) after the sample had been acid-digested in an Auto Kjeldahl digester system. Using a Soxhlet extraction heater (EAM-9201-06, Seoul, Republic of Korea), the crude lipid was measured following ether extraction (boiling point 40 °C–60 °C) [26].

### 2.5. Serum Analysis

Blood was collected from the caudal vein and was allowed to coagulate at room temperature for 30 min. Afterwards, it was centrifuged at a speed of 4000 rpm (1500 rcf, 8 × 5 mL Swing Out Rotor (BRK5508S), C1015 Micro Prime Centrifuge, Pocklington, UK) for 10 min at 4 °C. The pellet was discarded, and supernatant serum was transferred to new vials for further analysis.

#### 2.5.1. Total Serum Protein Estimation

Total protein was analyzed from the serum by a Randox Diagnostic (Cat. No. RX MONZA TP 245, Antrim, UK) kit that employs the Biuret method.

#### 2.5.2. Hormones Analysis

ELISA kit (CALBIOTECH, Cat. No. HG377S) was used for serum GH determination according to the manufacturer’s instructions. The optical density of each well was determined within 15 min using a microplate reader set at 450 nm. Based on the established standard curve, the GH concentration of each sample was determined. The thyroid stimulating hormone (TSH), triiodothyronine (T3), and thyroxine (T4) were measured in the supernatant serum using ELISA kits (Cat. No. DKO013, 125-300A, 225-300A, DiaMetra, Italy).

### 2.6. Analysis of Macromolecules

#### 2.6.1. Amino Acid Profile

Amino acids were determined by the method of Palliyeguru et al. [27] using an amino acid analyzer (Biochrom 30+, Biochrom Limited, Cambridge, UK). The 20 g of fish muscle was dried, of which 4 g was finely ground and oxidized with 500 mL of performic acid for methionine and cysteine conservation. The sample was hydrolyzed with 6M hydrochloric acid for 24 h, and the pH was adjusted to 2.2. The filtered sample was drenched in sample vials for the quantification of amino acids in the Biochrom 30+ amino acid analyzer by utilizing ion exchange chromatography.

#### 2.6.2. Fatty Acid Profile

The amount of 2 g of fish liver was homogenized in a blend of 1 mL of chloroform and 2 mL of methanol (*v*:*v*) by stirring for two minutes, collecting the bottom layer, and then removing the solvents using a nitrogen flow. The extraction was done twice, and the fatty acids profile of the fish was measured using a gas chromatography mass spectrometer (GC-MS) [28].

#### 2.6.3. Total Carbohydrate Content

For carbohydrates analysis, 2 g of muscle tissue was collected from each group and then homogenized. The resultant homogenate was transferred to Eppendorf tubes and, the total carbohydrate content was determined using SunLong Biotech Co., Ltd., Cat. No. AK08183-50T-48S, Hangzhou, China.

### 2.7. In Vivo Challenge Assay

*A. hydrophila* culture was prepared in 10 mL of nutrient broth (HiMedia Ltd., Lahore, Pakistan), vortexed, and then kept in a shaker incubator for 24 h at 37 °C. The culture was centrifuged at 8000 rpm (6100 rcf, 4 × 15 mL and 4 × 10 mL Small Fixed Angle Rotors (BRK5407S), C1015 Micro Prime Centrifuge, Pocklington, UK) for 15 min at 4 °C to get the hard pellet. The pellet was washed several times in sterile PBS and re-suspended in PBS (pH 7.4). The optical density of the bacterial suspension was measured using a UV spectrophotometer, and, finally, the culture was diluted with PBS to a corresponding concentration of 2 × 10^6^ CFU/mL [15].

Fish (*n* = 10) were placed randomly into four groups. The infected group (I) was intramuscularly injected with 0.5 mL (2 × 10^6^ CFU/mL) of *A. hydrophila* and given pelleted feed. The infected + probiotic-treated group (I + PL) was intramuscularly injected with *A. hydrophila* and treated with an optimal concentration (1.5 × 10^10^ CFU/kg feed) of the probiotic. The optimal concentration was chosen from the growth trial. The control group (Con) was injected with an equal volume of 1% sterilized PBS and was given pelleted feed. The probiotic-treated group (PL) was injected with an equal volume of 1% sterilized PBS and was also given 1.5 × 10^10^ CFU/kg feed of probiotic-supplemented feed. Fish were monitored for 12 days post-infection (dpi), during which the survival rate was noted. Survival (%) of challenged fish was calculated as follows: (total number of fish that survived/total number of fish stocked) × 100 [25].

#### 2.7.1. Colony Forming Unit (CFU) Counting

At the end of the challenge assay, an equivalent volume of muscle tissue from all experimental groups was collected and suspended in PBS separately to compare the bacterial burden in each group. For bacterial growth, homogenate samples of these liquids were plated on nutrient agar plates and maintained at 37 °C. The number of colonies was counted after 24 h of incubation by using a digital colony counter (Model: AVI-35).

#### 2.7.2. Hematological Assays

For hematological analysis, blood was collected from the caudal vein on days 3, 6, and 12 after infection. As an anticoagulant, ethylenediaminetetraacetic acid was used. Hematological parameters: red blood cells count (RBCs), hemoglobin (Hb), hematocrit (Hct%), mean corpuscular hemoglobin concentration (MCHC), mean corpuscular volume (MCV), packed cell volume (PCV), platelets (PLT), and white blood cells count (WBCs) were measured using an automated blood analyzer (Sysmex KX-21, Kobe, Japan).

#### 2.7.3. Histopathological Analysis

For histological analysis, soft tissues from the gill, liver, and kidney were obtained. To prevent cellular autolysis, collected tissues were preserved in 10% buffered formalin for 24 h. Fixed tissues were dehydrated by exposing them to several grades of alcohol in ascending order for 15 to 20 min, and then they were preserved in absolute ethanol [29]. Following two rounds of 100% pure xylene immersion, tissues were subsequently embedded in paraffin wax. On a microtome, tissue sections of 6–7 mm thickness was cut (ERM-2301). Later, to remove extra wax, pieces were deparaffinized by washing with alcohol and xylene. The slides were mounted and stained using distyrene-plasticizer, xylene (DPX), and eosin-hematoxylin stains (H&E), respectively. Tissues were photographed using a digital camera fitted with an optical microscope (Optika B-150DB, Ponteranica, Italy).

### 2.8. Statistical Analysis

Mean ± SD are used to express data. Data were statistically tested using the Graph-Pad Prism program (Version: 9.4.1, San Diego, CA, USA). The difference between groups was compared using analysis of variance (ANOVA) followed by Tukey’s multiple comparison method as a post hoc test.

## 3. Results

### 3.1. Growth Performance

A significant increase in body weight gain (WG), protein efficiency ratio (PER), specific growth rate (SGR), and condition factor (k), which showed the relationship among body length (L) and body weight (W) was seen in the probiotics-supplemented groups (T1, T2, T3, and T4) at the end of the experimental period when compared to the control group on every fortnight (2, 4, 6, 8, 10, and 12 weeks). A significant decrease in feed conversion ratio (FCR) in comparison to the control (T0) group was recorded (Table 1).

### 3.2. Proximate Analysis and Total Serum Protein Estimation

The values of moisture, ash, crude fat, and crude protein were calculated at the end of the trial period of three months (Table 2). The values of moisture and ash were increased in the treated groups (T1, T2, T3, and T4) than in the control (T0). Moisture was highest in the T2 group, while ash was maximum in the T3 group. The highest value of fat was recorded in the T2 *L. rhamnosus* group. The percent values of crude protein were significantly high in the T1, T2, and T3 groups in comparison to the T0 group. Serum total protein content was significantly higher in all the treated groups than that in the control group (Table 2).

### 3.3. Quantification of Serum Growth Hormone and Thyroid Hormone

**Figure 1:**
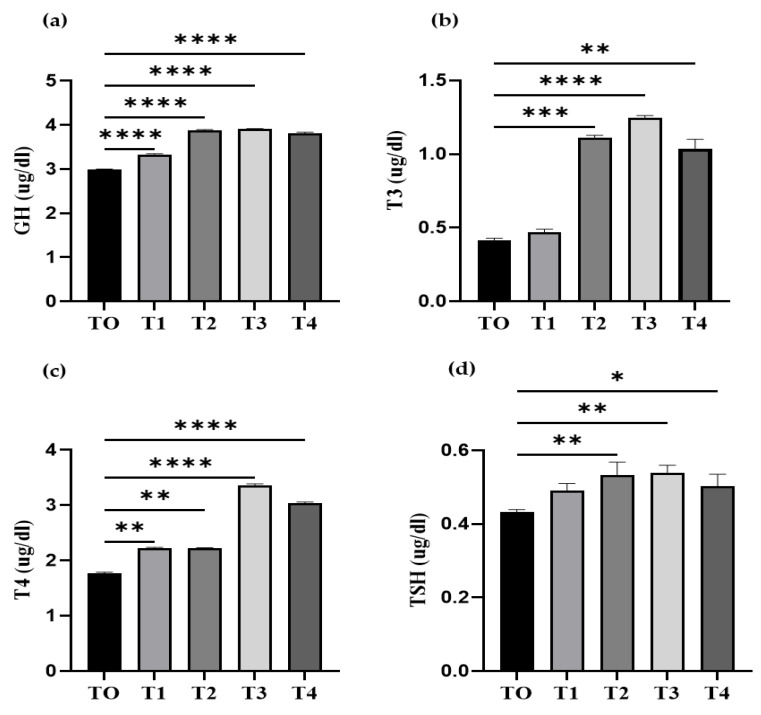
Quantification of serum growth and thyroid hormones. The quantity of growth and thyroid hormones in serum was determined at the end of the experimental trial of three months. (**a**) The concentration of growth hormone (GH), (**b**) the amount of Triiodothyronine (T3), (**c**) the amount of thyroxine (T4), and (**d**) the thyroid stimulating hormone (TSH). Data represent the mean ± SD of three independent replicates. Significant differences were examined using one-way ANOVA with a post hoc Tukey’s test, and asterisks indicate (* *p* < 0.05; ** *p* < 0.01, *** *p* < 0.001, and **** *p* < 0.0001) significant differences.

According to substantial (*p* < 0.05) polynomial responses over time (Figure 1a), the dietary inclusion of probiotics significantly increased the serum GH concentration. Serum GH levels increased considerably (*p* < 0.05) in a linear fashion in the treated groups. Fish that were fed T1, T2, T3, and T4 diets showed significantly higher (*p* < 0.05) GH concentrations than the T0-fed group. The effects of dietary inclusion of *L. rhamnosus* on the thyroid hormones of fish (Figure 1b–d) were significant in the T1, T2, T3, and T4 groups. Comparing the T2, T3, and T4 groups to the T0 group, the level of the hormone T3 increased significantly. In the T1, T2, T3, and T4 groups, the level of the T4 hormone increased significantly. The TSH level was significantly increased in T2, T3, and T4 groups.

### 3.4. Analysis of Macromolecules

#### 3.4.1. Amino Acid Profile

In the T0 and probiotic-fed groups, there was a substantial difference between the ten essential amino acids (EAA) and the nine non-essential amino acids (NEAA) (Table 3). Quantitatively, despite the statistically significant difference in the values, there was little variation between the mean values of the probiotic-fed groups and the T0 group. The probiotic groups had high EAA concentrations of cysteine, methionine, leucine, and histidine. Among NEAA, serine, glutamic acid, glycine, alanine, phenylalanine, and proline were found to be highest in the probiotic groups as compared to the T0 group. Overall, TNEAA levels were found to be higher than TEAA in the *L. rhamnosus*-treated groups.

#### 3.4.2. Fatty Acid Profile of Fish

Levels of palmitic acid and oleic acid were significantly high (*p* < 0.0001) in the probiotic-treated groups as compared to the T0 group (Table 4). Fish in the T0 groups showed the highest proportions of stearic acid (C_18:0_) (2.123%), linoleic acid (C_18:2_) (24.52%), and decosanoic acid (C_22:0_) (0.4457%) as compared to treatments. Decosahexanoic acid (C_22:6_) and eicosadionic acid (C_20:2_) were observed in T0, T1, T2, and T3. Eicosadionic acid was not observed in the T4 group (Table 4).

#### 3.4.3. Total Carbohydrate Content

**Figure 2:**
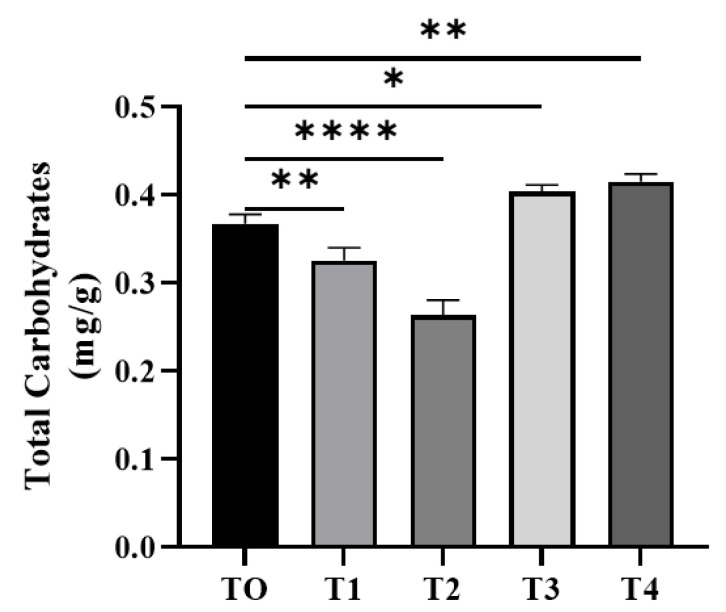
Estimation of muscle carbohydrate content. Data represent the mean ± SD of three replicates. Significant differences were examined using one-way ANOVA with a post hoc Tukey’s test, and asterisks indicate significant differences (* *p* < 0.05; ** *p* < 0.01, and **** *p* < 0.0001).

Carbohydrates values were significantly lower in the probiotic-treated groups T1 and T2 in comparison to the T0 group (0.3257 ± 0.008273 and 0.2637 ± 0.009711 in fish, respectively), and significantly higher values were recorded in the T3 and T4 groups (Figure 2).

### 3.5. In Vivo Challenge Assay

#### 3.5.1. Pathological Progression and CFU Analysis

**Figure 3:**
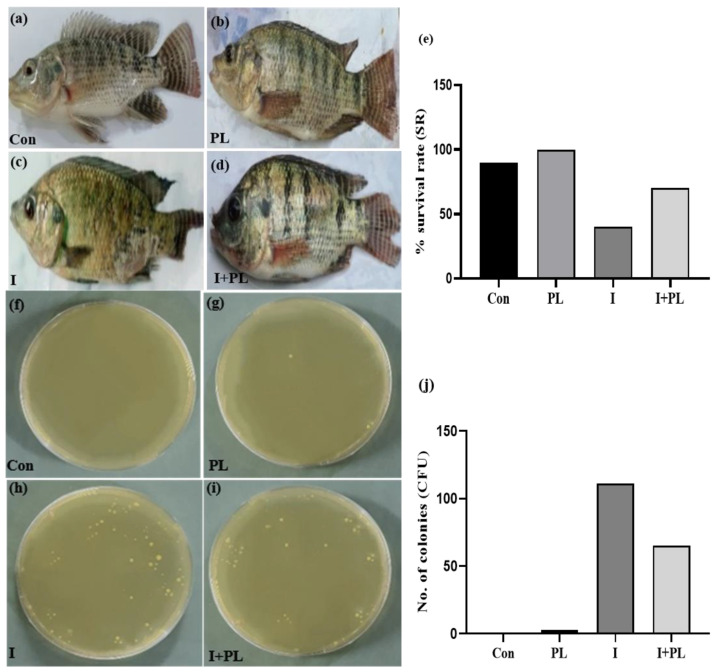
Propagation of *A. hydrophila* infection in fish; (**a**) control (Con) fish showing trunk region, group head, and tail region; (**b**) probiotic-treated (PL) fish showing gill lesions; (**c**) infected (I) fish showing redness of the eye, ulceration, scale loss, gill lesions, body swelling, tail rupturing, hemorrhage, dark spots, and surface bleeding; and (**d**) infected + probiotic-treated (I + PL) fish showing a change of body color, body swelling, tail rupturing, gill lesions. (**e**) The percent survival rate of fish. Bacterial burden was measured in fish muscle and the number of colonies was counted on agar plates; (**f**) control (Con), (**g**) probiotic-treated (PL) plate, (**h**) infected group (I), and (**i**) infected + probiotic-treated group (I + PL). The number of colonies was higher in the I group as compared to other treatments; (**j**) Histogram of CFU analysis.

After infection, fish were monitored for gross pathological changes. In the Con and PL groups, no pathological signs were observed in the whole body (Figure 3a,b). In the I group, fish indicated pathological signs starting at three dpi, notable eye redness, gill damage, hemorrhage, tail rupturing ulcerative lesions, trunk bleeding, scale loss, swelling, and red ulceration (Figure 3c). In the I + PL group, body color, body swelling, tail rupturing, and gill lesions were shown (Figure 3d). The survival rate of the I + PL group was high as compared to the I group after the *A. hydrophila* challenge. The Con and PL groups showed a higher survival rate than the challenge groups (Figure 3e). At the end of the challenge assay, the bacterial load was measured in fish muscle. Circular, smooth, and yellowish colonies of *A. hydrophila* were produced on nutrient agar, and the number of colonies was counted and found to be higher in the I group followed by the I + PL group in comparison to the Con and PL groups (Figure 3f–j).

#### 3.5.2. Hematological Indices

**Figure 4:**
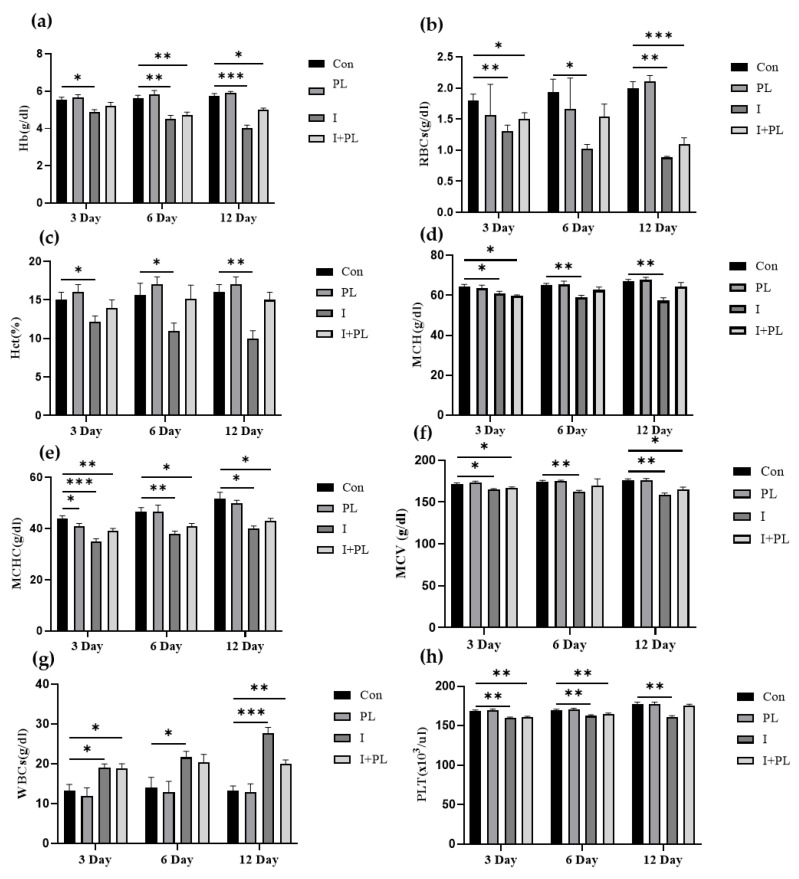
Hematological indices in control (Con), probiotic-treated (PL), infected (I), and infected + probiotic-treated group (I + PL) at 3, 6, and 12 dpi; (**a**) hemoglobin (Hb), (**b**) red blood cell count (RBCs), (**c**) hematocrit (Hct%), (**d**) mean corpuscular hemoglobin (MCH), (**e**) mean corpuscular hemoglobin concentration (MCHC), (**f**) mean corpuscular volume (MCV), (**g**) white blood cells (WBCs), and (**h**) platelets (PLT). Data are presented as mean ± SD (* *p* < 0.05; ** *p* < 0.01; and *** *p* < 0.001); tested using two-way ANOVA with post hoc Tukey’s test.

Hematological parameters were assessed after 3, 6, and 12 dpi. Significant differences (*p* < 0.05; *p* < 0.01; and *p* < 0.001) in Hb (Figure 4a), RBCs (Figure 4b), Hct (Figure 4c), MCH (Figure 4d), MCHC (Figure 4e), MCV (Figure 4f), and PLT (Figure 4h) were found in the I, and I + PL groups. However, WBC was significantly (*p* < 0.05) high in the I and I + PL groups as compared to the Con and PL groups (Figure 4g).

#### 3.5.3. Histopathological Examination

**Figure 5:**
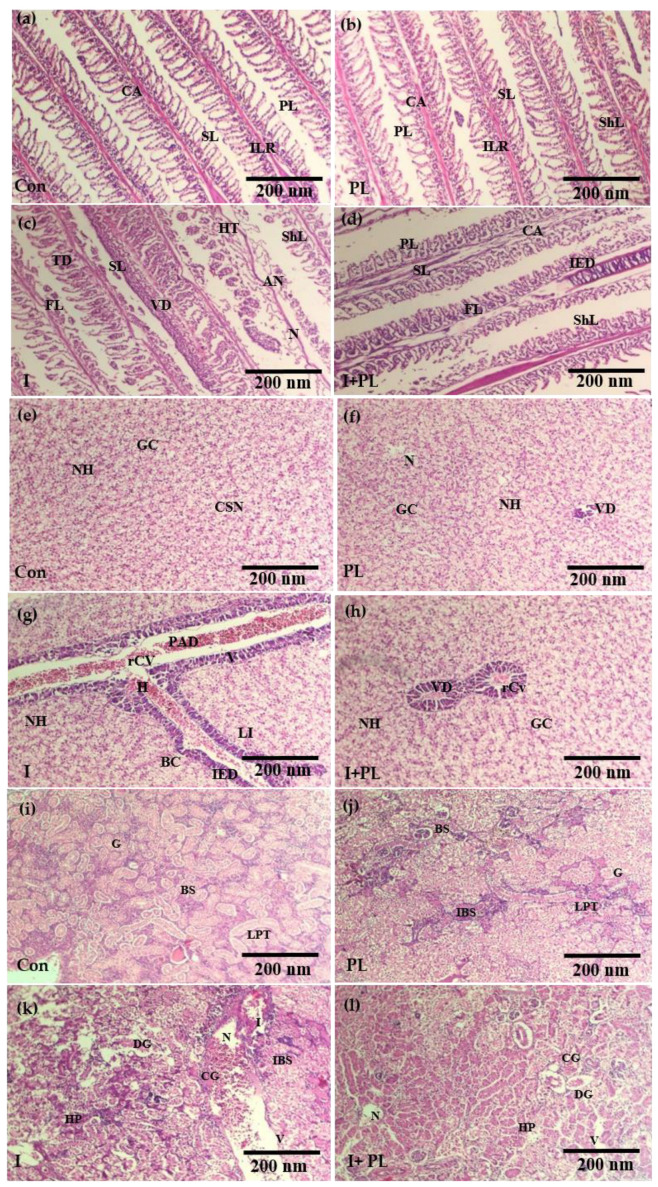
Gill tissues of control (Con), probiotic-treated (PL), infected (I), and infected + probiotic-treated (I + PL) fish (**a**), (**b**), (**c**), and (**d**), respectively; cellular necrosis (CN), fusion of lamellae (FL), primary lamellae (PL), secondary lamellae (SL), tissues debris (TD), aneurism (AN), interstitial oedema (IO), and shortening of lamellae (ShL). Liver tissues of control (Con), probiotic-treated (PL), infected (I), and infected + probiotic-treated (I + PL) fish (**e**), (**f**), (**g**), and (**h**), respectively; normal hepatocytes (NH), granular cytoplasm (GC), vacuolization of hepatocytes (V), central spheroidal hepatocyte nucleus (CSN), infiltration of oedematous fluid (IEF), cell necrosis (N), degeneration of pancreatic area (DPA), pyknotic nuclei (PN), cytoplasmic degeneration (CD), leukocytes infiltration (LI), rupturing of the central vein (rCV), and hyperplasia (HP). Kidney tissues of Con, probiotic-treated (PL), infected (I), and infected + probiotic-treated (I + PL) fish (**i**), (**j**), (**k**), and (**l**), respectively; glomeruli (G), degenerated glomeruli (DG), Bowman’s space (BS), constricted glomeruli (CG), increased Bowman’s space (IBS), longitudinal section of proximal tubule (LPT), necrosis (N), diffuse exudation of erythrocytes (DE) hypertrophied renal tubule (HRT), hemopoietic tissue (H), vacuolization (V), hyperplasia (HP), and H&E stained, 10X. Scale bar = (**a**–**l**): 200 nm.

The histopathological changes observed after 12 dpi in the gills of the Con group revealed distinctive structures of epithelial cells lining lamellae; a rod-like axis found at the middle of primary gill lamellae was organized around both sides of secondary gill lamellae, and similar changes were observed in the PL group (Figure 5a,b). The variations in gill histology in the I group indicated hemorrhage, curling, shortening of lamellae, cyto-architectural alteration, hypertrophy, cellular necrosis, vasodilation, and lamellar epithelial rupture. A thin epithelial cell layer of secondary gill lamella at their distal end was free (Figure 5c). The gills of the I + PL groups showed intracellular edema, cellular necrosis, fusion of lamellae, and shortening and curling of the lamellae (Figure 5d).

In the liver of Con fish, no histological changes were observed. Hepatocytes, with spherical nuclei and polygonal shapes arranged in different tubules, were identified (Figure 5e). The PL group showed vasodilation in the sinusoids, necrosis, cytoplasmic degeneration, and infiltration of edematous fluid (Figure 5f). The severe pathological changes observed in the I group were caused by *A. hydrophila* in the liver of the nuclei and severe dilation in sinusoids with lipid vacuoles, which caused pyknotic nuclei, cytoplasmic degeneration, intravascular, hemolysis, and hemosiderin accumulation. Moreover, coagulate-type cellular necrosis, congestion of sinusoid hypertrophy, cytoplasmic hyperplasia, and vacuolization were also seen (Figure 5g). In the I + PL group, histological alterations were observed, including leukocyte infiltration, melano-macrophage aggregation, hypertrophy, hyperplasia, and hemosiderin accumulation. Variations in tissue structure, such as rupturing of the central vein, were also observed (Figure 5h).

Histomorphological examination of the Con group kidney indicated structure nephrons, Bowman’s capsule, proximal, distal, and collecting tubules (Figure 5i). In the PL group, increased Bowman space and minor alterations in the longitudinal section of the proximal tubule were observed (Figure 5j). In the I group, pathological changes of the kidney indicated interstitial hemorrhage, hemosiderin accumulation, increased Bowman’s space, constricted glomeruli, hyperplasia, vacuolization, massive atrophy of the renal tubule, aggregation of inflammatory cells, hypertrophied renal tubule, and massive erythrocytes, which were diffusely exuding while edematous fluid was infiltrating (Figure 5k). The kidney structure of the I + PL groups showed a variable longitudinal section of the proximal tubule, degenerated glomeruli, hyperplasia, increased Bowman’s space, vacuolization, and necrosis (Figure 5l).

## 4. Discussion

In aquaculture, probiotics are recognized as eco-friendly microbial control agents. They not only aid in the fight against diseases but also enhance fish growth and survival through improved feed utilization [30,31]. The probability of harmful bacterial infections is decreased when probiotic microorganisms colonize the gut of animals. This promotes the health of organisms enabling them to fight against different diseases [32,33]. The goal of this study was to identify the unique advantages of employing probiotics in the feed of *O. niloticus* from the standpoint of growth performance as well as their role in the immune response of treated fish to pathogenic bacteria, *A. hydrophila.*

A probiotic (*L. rhamnosus*) was employed for three months with four different concentrations, after which growth parameters were evaluated. The findings indicated substantial differences in different parameters in the treated and control group. In a diet supplemented with 1.5 × 10^10^ CFU/kg concentration, fish grew fastest and showed the highest SGR. The probiotic-supplemented diets showed a better condition factor than those in the control group. Among the groups receiving the probiotic-supplemented diets, only fish kept on a basal diet had a greater FCR than those in the probiotic-treated groups. A lower FCR indicates better fish feed consumption, which is a key measure of the quality of fish diets [34]. Fish fed on Biogen^®^ showed a 33% rise in their daily growth rate and a 43% decrease in their FCR [35].

Probiotics have been reported to reduce the amount of feed required for animal growth, thereby lowering the production costs for the farmers [36]. The PER results showed that supplementing meals with probiotics had a good impact, as seen by the high PER with low fat levels, which dramatically increased the ability of *O. niloticus* to use protein. Protein is the most expensive nutrient for growth, and probiotics help to maximize the use of protein. Probiotic supplements improve protein utilization in stressful situations, by the enhancement in the biological value of supplemented diets [37].

The percentage of crude protein, crude fats, and ash in probiotic-treated groups was statistically significant as compared to the control group. Abdel-Tawwab et al. [38] determined that S. cerevisiae are essential for improving fish body composition by increasing food intake. As a result, the increased carcass protein might be attributed to the probiotics in the Nile tilapia gut secreting more proteins and the efficient conversion of the food that helps fish build more muscle. In a recent study, Azarin et al. [39] reported greater protein-lipid, moisture, and ash levels in *L. rohita* fed with *B. subtilis* and *B. circulans*-supplemented feed. The total protein is a good indicator of an organism’s health and natural defenses. The total protein content of serum from the probiotics-fed groups was significantly higher than that of the control group. This is consistent with earlier research showing that probiotics increase the amount of total protein in catla [40].

GH performs vital functions in the body including growth, immune regulation, and functions of behavioral activities [41,42,43]. The probiotic supplementation increased serum GH levels significantly. Adding a probiotic (*Enterococcus faecium*) to the water during the rearing of clownfish caused unexpectedly increased expression of GH, which was connected with a threefold increase in body weight compared to the control group [44]. To the best of our knowledge, the current work is the first to evaluate the growth potential of *O. niloticus* with *L. rhamnosus* supplementation.

Thyroid hormones play a significant role in a variety of physiological processes including development, growth, behavior, and stress [45,46]. We found an increase in T3, T4, and TSH levels in the probiotic-treated groups. Thyroid hormones were markedly enhanced following prebiotic administrations [47]. Numerous studies have shown that thyroid hormones have metabolic functions that are strongly correlated with factors that promote growth [48]. Due to the potential for TSH levels in the blood to serve as a reliable predictor of thyroid health, monitoring TSH levels aids in the management of thyroid illnesses [49]. Altered levels of thyroid hormone in fish might cause anomalies in their neurological and metabolic systems [50].

Amino acids are crucial for the body’s natural healing processes, and since fish and humans have comparable amino acid compositions, eating fish can provide people with a balanced diet of all the essential amino acids. A lack of necessary amino acids may obstruct the repair and recovery process [51]. Our results showed that *O. niloticus* carries all nine necessary amino acids. Probiotic-treated fish showed a comparatively high amount of essential and non-essential amino acids as compared to non-treated fish [52]. Bone, skin, and muscle tissue can repair faster due to leucine. Isoleucine is important for the synthesis of hemoglobin, blood sugar regulation, and energy stabilization. Along with other necessary amino acids such as alanine, glycine, one of the primary components of human skin collagen, promotes tissue repair and regeneration [53].

Supplementation of probiotics showed higher levels of palmitic acid and oleic acid in comparison to the untreated control group. The highest levels of C_18:0_, C_18:2_, and C_22:0_ were observed in the control group. Moreover, high DHA levels in the liver samples of the probiotic-treated groups suggested that some ALA had been converted into n-3 LC polyunsaturated fatty acids. It implies that the ability of carp to desaturate and extend ALA to EPA and DHA exists. Similar findings have been noted in several freshwater fish species, including trout and common carp [54,55].

Carbohydrate values were significantly lower in probiotic-treated groups. A study on *Pseudotolihus. typus* and *P. elongatus* showed that the carbohydrate content was low [56]. The difference in composition might be due to the different species and different environmental conditions.

Phenotypic examination of infected *O. niloticus* showed general septicemia signs [57,58]. The type of host, the organism’s age, and the stage of sickness, whether acute or chronic, all have an impact on the clinical signs that any infection may induce [59]. It has been observed that Pseudomonas sp. and *Aeromonas* sp. are responsible for ulcer-type infections in freshwater fish [60,61].

The Con and probiotic-administered groups showed higher percent survival rates than the infected. These findings are consistent with Kumar et al. [62], who found that *L. rohita* had a high survival rate in the *B. subtilis*-supplemented group. Parallel to the phenotypic data, the number of bacterial colonies were less in the infected fish treated with *L. rhamnosus* than the untreated infected fish.

Hemoglobin concentration and erythrocyte count significantly decreased in the challenged group as compared to the control group, at 3, 6, and 12 dpi. Considerable reduction in the total erythrocyte count, hemoglobin content, hematocrit, and reduced erythrocyte diameter has been previously reported in fish after the *A. hydrophila* challenge [63,64]. The movement of hypochromic erythrocytes from the spleen towards other hematopoietic organs is the cause of a decrease in RBC and hemoglobin levels [65]. The leukocyte count was significantly high in the infected group. *A. hydrophila* infection had a similar impact on the leukocyte count in African catfish [66].

Histopathology is employed to analyze tissue damage caused by various chemicals or biological infectious agents [67,68]. Gills are especially vulnerable to aquatic infections because they are constantly in contact with the outside environment [69]. Histological deformities were abundant in the gills of the infected group as compared to the treated group. Erythrocyte congestion consequently occurred in the marginal channel [70]. The liver histology of the probiotic-treated group exhibited traits resembling those of the Con. The inability of the liver to properly remove foreign particles causes hepatocyte degeneration and congestion in the sinusoids [71]. The presence of extracellular toxins produced by *A. hydrophila* may be the cause of lipid vacuoles and necrosis in the liver [72,73]. Similar hepatic lesions, e.g., lymphocyte infiltration, focal necrosis, and cytoplasmic fat vacuoles have also been observed in other species, including carp [74]. Kidney tissues of fish infected with *A. hydrophila* showed severe cellular necrosis and glomerular changes. The glomerular epithelium in the kidney of a catfish that had been infected with *A. hydrophila* had histological alterations [75]. The histopathology results support and confirm our examined hematological parameters and are consistent with the previous findings of the pathological effects of *Aeromonas* infection in *O. niloticus*.

## 5. Conclusions

The findings of this study show that *L. rhamnosus* had a beneficial impact on *O. niloticus* growth, proximate composition, thyroid hormones, hematological, and immunological indices, as well as survival against *A. hydrophila* infection. Our findings suggest that 1.5 × 10^10^ CFU/kg was the optimal concentration for improving *O. niloticus* growth and health. The use of probiotics as a safe substitute for antimicrobial agents can be suggested in light of these findings. We believe that our results have important implications for the aquaculture industry, as they provide a sustainable and eco-friendly approach to improve fish production and quality.

## Figures and Tables

**Table 1:** The growth parameters of the fish measured at the end of the growth trial (three months).

Parameters		Treatments				
		T0	T1	T2	T3	T4
W	Initial	20.06 ± 0.089	20.02 ± 0.044	20.06 ± 0.054	20.02 ± 0.044	20.06 ± 0.054
	2 Weeks	21.10 ± 0.787	23.02 ± 0.438 *	23.32 ± 0.455 **	23.90 ± 0.430 **	23.34 ± 0.482 **
	4 Weeks	24.08 ± 0.396	25.20 ± 0.495 *	25.98 ± 0.775 *	27.26 ± 1.085 **	26.50 ± 1.000 *
	6 Weeks	25.30 ± 0.721	28.80 ± 0.908 **	30.60 ± 1.140 ***	33.36 ± 1.270 ****	31.98 ± 0.746 ****
	8 Weeks	27.40 ± 0.547	30.40 ± 3.362 *	34.80 ± 1.304 ****	37.36 ± 1.717 ****	36.96 ± 0.712 ****
	10 Weeks	29.00 ± 1.225	38.80 ± 1.924 ***	39.60 ± 1.140 ****	41.40 ± 1.140 ****	39.20 ± 1.095 ****
	12 Weeks	32.00 ± 1.581	44.20 ± 1.789 ****	45.40 ± 1.140 ****	48.00 ± 1.581 ****	45.20 ± 0.836 ****
L	Initial	10.06 ± 0.054	10.04 ± 0.054	10.08 ± 0.044	10.06 ± 0.054	10.02 ± 0.044
	2 Weeks	10.24 ± 0.114	10.38 ± 0.044	10.42 ± 0.083	10.50 ± 0.122 *	10.42 ± 0.083
	4 Weeks	10.72 ± 0.130	10.92 ± 0.148	11.16 ± 0.219 *	11.62 ± 0.277 **	11.60 ± 0.223 **
	6 Weeks	11.32 ± 0.216	12.04 ± 0.384 **	12.06 ± 0.336 **	12.36 ± 0.296 ****	12.20 ± 0.264 ****
	8 Weeks	12.20 ± 0.291	13.06 ± 0.270 ****	13.30 ± 0.234 ****	13.82 ± 0.083 ****	13.56 ± 0.296 ****
	10 Weeks	12.72 ± 0.178	13.92 ± 0.192 ****	14.66 ± 0.181 ****	14.90 ± 0.141 ****	14.62 ± 0.277 ****
	12 Weeks	13.18 ± 0.204	15.18 ± 0.249 ****	15.66 ± 0.260 ****	15.98 ± 0.083 ****	15.84 ± 0.114 ****
WG	2 Weeks	1.100 ± 0.787	3.000 ± 0.403 *	3.260 ± 0.456 **	3.880 ± 0.396 **	3.300 ± 0.489 **
	4 Weeks	4.020 ± 0.389	5.180 ± 0.521 *	5.920 ± 0.746 *	7.240 ± 1.101 **	6.460 ± 0.950 *
	6 Weeks	5.240 ± 0.650	8.380 ± 1.359 *	10.54 ± 1.137 ***	13.34 ± 1.305 ***	11.94 ± 0.740 ****
	8 Weeks	6.940 ± 1.307	12.38 ± 2.043 *	15.30 ± 1.875 ***	17.78 ± 1.481 ***	16.52 ± 1.130 ****
	10 Weeks	8.940 ± 1.307	18.78 ± 1.921 ***	19.54 ± 1.180 ****	21.38 ± 1.150 ****	19.16 ± 1.106 ****
	12 Weeks	11.94 ± 1.599	24.18 ± 1.764 ****	25.34 ± 1.167 ****	27.98 ± 1.550 ****	25.16 ± 0.820 ****
LG	2 Weeks	0.180 ± 0.109	0.340 ± 0.054	0.340 ± 0.547	0.440 ± 0.167 *	0.400 ± 0.070
	4 Weeks	0.660 ± 0.114	0.880 ± 0.164	1.080 ± 0.216 *	1.560 ± 0.260 **	1.380 ± 0.295 *
	6 Weeks	1.280 ± 0.204	2.000 ± 0.367 *	1.980 ± 0.334 *	2.300 ± 0.291 **	2.180 ± 0.295 **
	8 Weeks	2.140 ± 0.270	3.020 ± 0.228 **	3.220 ± 0.216 ***	3.760 ± 0.114 ***	3.540 ± 0.288 ***
	10 Weeks	2.660 ± 0.134	3.880 ± 0.228 ***	4.660 ± 0.151 ****	4.720 ± 0.238 ****	4.660 ± 0.320 ****
	12 Weeks	3.120 ± 0.164	5.140 ± 0.219 ****	5.580 ± 0.286 ****	5.920 ± 0.083 ****	5.820 ± 0.109 ****
SGR	2 Weeks	0.150 ± 0.104	0.403 ± 0.054 *	0.408 ± 0.057 **	0.495 ± 0.032 **	0.361 ± 0.173
	4 Weeks	0.265 ± 0.020	0.332 ± 0.029 *	0.373 ± 0.041 *	0.449 ± 0.054 **	0.403 ± 0.050 *
	6 Weeks	0.225 ± 0.026	0.336 ± 0.046 *	0.406 ± 0.036 ***	0.492 ± 0.039 ****	0.450 ± 0.022 ****
	8 Weeks	0.215 ± 0.033	0.361 ± 0.057 *	0.401 ± 0.029 ***	0.459 ± 0.028 ****	0.434 ± 0.022 ****
	10 Weeks	0.213 ± 0.024	0.382 ± 0.028 ****	0.393 ± 0.018 ****	0.420 ± 0.016 ****	0.387 ± 0.016 ****
	12 Weeks	0.225 ± 0.023	0.381 ± 0.019 ****	0.393 ± 0.012 ****	0.421 ± 0.015 ****	0.391 ± 0.008 ****
FCR	2 Weeks	4.126 ±1.206	2.018 ± 0.303	1.856 ± 0.295	1.742 ± 0.330 *	1.842 ± 0.277
	4 Weeks	2.792 ± 1.059	2.340 ± 0.219	2.040 ± 0.270	1.680 ± 0.238	1.860 ± 0.251
	6 Weeks	3.478 ± 0.456	2.160 ± 0.350 **	1.700 ± 0.200 **	1.380 ± 0.192 ***	1.500 ± 0.100 **
	8 Weeks	3.578 ± 0.825	1.980 ± 0.334 *	1.600 ± 0.141 *	1.340 ± 0.114 *	1.440 ± 0.114 *
	10 Weeks	3.420 ± 0.575	1.580 ± 0.148 **	1.520 ± 0.083 **	1.400 ± 0.070 **	1.540 ± 0.054 **
	12 Weeks	3.026 ± 0.448	1.500 ± 0.100 **	1.400 ± 0.070 **	1.320 ± 0.083 **	1.420 ± 0.044 **
PER	2 Weeks	3.620 ± 2.639	9.960 ± 1.431 *	10.82 ± 1.527 **	12.90 ± 1.319 **	29.76 ± 4.371 ****
	4 Weeks	13.34 ± 1.299	17.22 ± 1.756 *	19.72 ± 2.508 *	24.10 ± 3.673 **	22.78 ± 3.627 *
	6 Weeks	17.42 ± 2.163	27.88 ± 4.549 *	35.08 ± 3.789 ***	44.42 ± 4.356 ***	39.78 ± 2.480 ****
	8 Weeks	22.98 ± 4.214	41.22 ± 6.796 **	49.84 ± 4.775 ***	59.22 ± 4.928 ****	55.06 ± 3.764 ****
	10 Weeks	29.76 ± 4.371	62.58 ± 6.406 ***	65.10 ± 3.948 ****	71.22 ± 3.832 ****	63.82 ± 3.675 ****
	12 Weeks	39.78 ± 5.320	80.56 ± 5.876 ****	84.44 ± 3.911 ****	93.22 ± 5.156 ****	83.84 ± 2.712 ****
CF	2 Weeks	1.990 ± 0.731	1.636 ± 0.848	2.078 ± 0.089	2.046 ± 0.040	2.058 ± 0.066
	4 Weeks	1.890 ± 0.022	1.910 ± 0.103	1.934 ± 0.060	1.964 ± 0.047 *	1.956 ± 0.102
	6 Weeks	1.564 ± 0.104	1.768 ± 0.092	1.906 ± 0.021 **	1.868 ± 0.084 **	1.802 ± 0.102 *
	8 Weeks	1.388 ± 0.052	1.642 ± 0.207 *	1.580 ± 0.094	1.606 ± 0.102 *	1.512 ± 0.075
	10 Weeks	1.342 ± 0.037	1.514 ± 0.072	1.528 ± 0.082 *	1.616 ± 0.100 **	1.526 ± 0.068 *
	12 Weeks	1.310 ± 0.053	1.396 ± 0.080	1.512 ± 0.132	1.540 ± 0.0961 *	1.544 ± 0.078 **

Data are presented as mean ± SD (* *p* < 0.05; ** *p* < 0.01; *** *p* < 0.001; and **** *p* < 0.0001); tested using two-way ANOVA with post hoc Tukey’s test.

**Table 2:** Effects of *L. rhamnosus* on the biochemical composition of *O. niloticus*.

Parameters	T0	T1	T2	T3	T4
Moisture (%)	73.30 ± 0.360	74.17 ± 1.258	74.73 ± 1.617	73.80 ± 0.200	74.03 ± 0.450
Ash (%)	2.057 ± 0.125	2.263 ± 0.257	2.470 ± 0.036	2.537 ± 0.035 *	2.430 ± 0.130
Fat (%)	5.133 ± 0.152	5.947 ± 0.382	6.500 ± 0.264 *	5.533 ± 0.152	6.467 ± 1.002 *
Protein (%)	63.75 ± 2.308	71.39 ± 1.451 **	70.43 ± 0.684 **	68.78 ± 2.335 *	64.10 ± 2.007
Serum total protein (g/dl)	2.027 ± 0.064	2.130 ± 0.020 **	2.240 ± 0.030 ***	2.823 ± 0.027 ****	2.643 ± 0.025 ****

Values are presented as mean ± SD. Mean with different superscript asterisks in a row are statistically significant at (* *p* < 0.05; ** *p* < 0.01, *** *p* < 0.001, and **** *p* < 0.0001); tested using one-way ANOVA with post hoc Tukey’s test.

**Table 3:** Analyzing the essential and non-essential amino acids in muscle samples from the control group and the *L. rhamnosus*-treated groups.

Amino Acids	Treatments				
EAA (%)	T0	T1	T2	T3	T4
Cysteine	0.500 ± 0.010	0.563 ± 0.025	0.620 ± 0.052 **	0.600 ± 0.036 **	0.603 ± 0.005 **
Methionine	1.623 ± 0.020	1.657 ± 0.030	1.667 ± 0.037	1.710 ± 0.020 *	1.717 ± 0.032 **
Threonine	2.477 ± 0.453	2.253 ± 0.026	2.640 ± 0.606	2.657 ± 0.617	2.337 ± 0.047
Valine	2.283 ± 0.011	2.313 ± 0.033	2.350 ± 0.026	2.367 ± 0.025 *	2.350 ± 0.040
Isoleucine	2.170 ± 0.020	2.180 ± 0.010	2.177 ± 0.030	2.207 ± 0.028	2.160 ± 0.036
Leucine	3.543 ± 0.041	3.577 ± 0.021	3.653 ± 0.035 **	3.667 ± 0.031 **	3.620 ± 0.020 *
Histidine	1.340 ± 0.062	1.453 ± 0.025 *	1.430 ± 0.026	1.423 ± 0.051	1.377 ± 0.032
Lysine	3.937 ± 0.040	3.930 ± 0.017	3.943 ± 0.035	3.923 ± 0.022	3.887 ± 0.011
Arginine	3.207 ± 0.100	3.310 ± 0.015	3.260 ± 0.043	3.247 ± 0.055	3.317 ± 0.045
Non-EAA (%)	**T0**	**T1**	**T2**	**T3**	**T4**
Aspartic Acid	3.637 ± 0.032	3.647 ± 0.049	3.683 ± 0.058	3.697 ± 0.035	3.670 ± 0.020
Serine	2.287 ± 0.015	2.373 ± 0.030 *	2.557 ± 0.025 ****	2.520 ± 0.030 ****	2.490 ± 0.040 ****
Glutamic Acid	7.450 ± 0.040	7.570 ± 0.043 **	7.707 ± 0.021 ****	7.687 ± 0.035 ****	7.657 ± 0.038 ***
Glycine	3.480 ± 0.026	3.563 ± 0.030 *	3.553 ± 0.045	3.563 ± 0.031 *	3.513 ± 0.032
Alanine	3.163 ± 0.015	3.087 ± 0.041 *	3.220 ± 0.020	3.297 ± 0.025 ***	3.250 ± 0.030 *
Phenylalanine	2.160 ± 0.026	2.193 ± 0.011	2.190 ± 0.010	2.243 ± 0.015 **	2.217 ± 0.021 *
Tyrosine	1.263 ± 0.025	1.297 ± 0.021	1.293 ± 0.040	1.320 ± 0.020	1.280 ± 0.030
Proline	2.600 ± 0.050	2.633 ± 0.015	2.767 ± 0.065 **	2.710 ± 0.034 *	2.620 ± 0.030
Ornithine	0.156 ± 0.035	0.216 ± 0.056	0.226 ± 0.032	0.176 ± 0.034	0.170 ± 0.026

Values are presented as mean ± SD. The mean with superscript asterisks in a row is statistically significant at (* *p* < 0.05; ** *p* < 0.01, *** *p* < 0.001, and **** *p* < 0.0001); tested using one-way ANOVA with post hoc Tukey’s test.

**Table 4:** Comparison of the fatty acid profile (%) in total liver lipids of the control and the *L. rhamnosus*-treated groups.

Carbon No.	Fatty Acids	T0	T1	T2	T3	T4
C_14:0_	Myristic acid	0.436 ± 0.030	0.216 ± 0.006 ***	0.136 ± 0.021 ***	0.726 ± 0.025 ***	0.463 ± 0.020
C_16:0_	Palmitic acid	24.68 ± 0.476	31.95 ± 0.832 ****	38.41 ± 0.364 ****	28.31 ± 0.305 ****	31.33 ± 0.115 ****
C_17:0_	Heptadecanoic acid	0.913 ± 0.015	0.710 ± 0.017 ****	1.263 ± 0.030 ****	0.536 ± 0.021 ****	0.770 ± 0.026 ****
C_18:0_	Stearic acid	2.123 ± 0.032	0.913 ± 0.035 ****	1.207 ± 0.040 ****	0.510 ± 0.026 ****	1.267 ± 0.025 ****
C_18:1_	Oleic acid	35.54 ± 0.134	39.53 ± 0.464 ****	36.08 ± 0.030 ****	33.03 ± 0.049 ****	33.19 ± 0.043 ****
C_18:2_	Linoleic acid	24.52 ± 0.453	19.77 ± 0.091 ****	12.64 ± 0.106 ****	16.31 ± 0.337 ****	19.87 ± 0.585 ****
C_20:2_	Eicosadionic acid	1.553 ± 0.045	0.560 ± 0.030 ****	0.783 ± 0.031 ****	1.330 ± 0.034 ****	-
C_22:0_	Decosanoic acid	0.193 ± 0.035	0.186 ± 0.023	-	0.176 ± 0.040	0.126 ± 0.028
C_22:6_	Decosahexanoic acid	0.446 ± 0.015	0.276 ± 0.032 ****	0.376 ± 0.030 *	0.213 ± 0.025 ****	0.373 ± 0.028 *

Values are presented as mean ± SD. The mean with superscript asterisks in a row is statistically significant at (* *p* < 0.05, *** *p* < 0.001, and **** *p* < 0.0001); tested using one-way ANOVA with post hoc Tukey’s test.

## Data Availability

The data will be available from the corresponding author upon request.
